# Cardiac MRI for differentiating chemotherapy-induced cardiotoxicity in sarcoma and breast cancer

**DOI:** 10.2478/raon-2025-0012

**Published:** 2025-02-27

**Authors:** El-Sayed H Ibrahim, Lubna Chaudhary, Yee-Chung Cheng, Antonio Sosa, Dayeong An, John Charlson

**Affiliations:** 1 Medical College of Wisconsin, Milwaukee, USA; 2 Northwestern University, Evanston, USA

**Keywords:** cardiotoxicity, MRI, chemotherapy, sarcoma, breast cancer

## Abstract

**Background:**

Over the past few decades, many studies have focused on anthracyclines effect on the heart (cardiotoxicity), but only a few have focused on sarcoma. In this study, we harness the capabilities of advanced cardiac magnetic resonance imaging (MRI) for characterizing anthracyclines-induced cardiotoxicity in sarcoma and compare the results to those from breast cancer patients.

**Patients and methods:**

The patients receive an MRI exam at three timepoints: baseline (pre-treatment), posttreatment, and at 6-months follow-up.

**Results:**

The results demonstrated a differential response in sarcoma, characterized by increasing left-ventricular (LV) mass and decreasing right ventricular ejection fraction (RVEF). In all patients, left ventricular ejection fraction (LVEF) remained > 50% at all timepoints. Myocardial strain was always lower than the normal threshold values and showed small changes between different timepoints. Myocardial T2 and extracellular volume (ECV) showed increasing and decreasing patterns, respectively, in sarcoma, which were the opposite patterns of those in breast cancer. While myocardium T1 showed increasing values in breast cancer, T1 in sarcoma increased post-treatment and then decreased at the 6-months follow-up. The results showed inverse correlation between dose and different strain components in sarcoma, which was not the case in breast cancer. Certain myocardial segments showed high correlation coefficients with dose, which may reflect their increased sensitivity to cardiotoxicity.

**Conclusions:**

Cardiac MRI proved to be a valuable technique for determining anthracycline-induced changes in cardiac function and myocardial tissue composition in sarcoma and differentiating it against breast cancer. It also provides a comprehensive assessment of heart health at baseline, which is important for risk stratification.

## Introduction

Despite being rare in the general population, sarcoma is the second most common cancer among children and young adults. Sarcoma tumors originate from mesenchymal cells in different body areas.^1^ The number of cancer survivors has significantly increased over the past few decades due to treatment improvements.^2^ Despite the development of advanced cancer therapies, anthracyclines remain a commonly used treatment for cancer, including sarcomas and breast cancer.^3^ In particular, doxorubicin is considered a standard, first-line treatment of sarcoma.^4^ Nevertheless, treatment with doxorubicin has the side effect of cardiotoxicity with potential development of heart failure if not promptly treated.^5^ Sarcoma patients are more likely than other cancer patients to develop cardiotoxicity after receiving anthracyclines.^6^ In pediatric sarcoma patients enrolled in the prospective Late Effects Surveillance System study, which included 265 patients, the cardiotoxicity incidence was 7.5%.^7^ Another registry showed a 14% incidence of cardiotoxicity in 43 adult patients.^8^

Early onset cardiotoxicity is a major challenge in clinical practice due to the reliance on anthracyclines and relative lack of other systemic therapy options for treatment of advanced sarcomas.^3^ Cardiotoxicity is defined by the development of heart failure symptoms or by asymptomatic decrease in baseline left ventricular ejection fraction (LVEF) ≥ 10% to a level < 50%.1,9,10 Cardiotoxicity can cause cardiovascular complications, including ventricular dysfunction, myocardial ischemia, hypertension, arrhythmias, and heart failure.^1–14^ A potential mechanism of cardiotoxicity development is myocyte free radical damage, which is emphasized by repetitive damage to myocyte mitochondria and high peak levels of plasma.^15,16^ Cardiotoxicity has been well studied among patients with breast cancer^8,17^; however, there is limited data regarding cardiotoxicity and mortality rates in sarcoma patients.^18^ The treatment and prognosis of sarcoma and breast cancer are different, and using results from breast cancer studies to interpret those from sarcomas may not be appropriate. Anthracyclines induced cardiotoxicity can occur after both high- and low-dose doxorubicin therapy^19^ due to wide variation in individual vulnerability^20^ with no clear threshold regarding safe doses of anthracyclines.^21,22^ Actually, the cumulative dosage of anthracyclines used in sarcoma is higher than that in many other cancers^23^ as using doxorubicin beyond the recommended cumulative dose is a promising option to improve survival in patients with advanced sarcomas.^24^ Although longterm surveillance guidelines of cancer patients receiving anthracyclines are addressed in the literature, there is no clear guidelines regarding surveillance during and shortly after treatment.^25^

In clinical practice, a decrease in LVEF is the most common form of cardiotoxicity.^5,26^ However, a large reduction in LVEF occurs late in the process of cancer therapy induced cardiotoxicity.^27^ Therefore, it is important to stratify patients early on such that cardiac protection can be initiated as early as possible in those with increased risk of cardiotoxicity. It has been shown that MRI global longitudinal strain (GLS) is more sensitive than LVEF for detecting early signs of systolic myocardial dysfunction.^22,28^ GLS may also identify patients at risk of cardiotoxicity, possibly through detection of baseline subclinical cardiac dysfunction, which may advance to heart failure.^29^ Either a low absolute GLS value early during chemotherapy or a threshold relative reduction in GLS compared with baseline can be used to identify individuals at high risk of developing heart failure.^29^ GLS less than 17% (in absolute value) or relative reduction in GLS by >15% from baseline has been used as a threshold for patients at risk.^27,30^ Changes in other strain components, *e.g*., global circumferential strain (GCS), have been also reported in sarcoma.^31^

It has been reported that patients with subsequent cardiotoxicity may have low myocardial T1 times and decreased LV mass; but patients do not typically develop myocardial scars or focused fibrosis from anthracyclines.^32^ Nevertheless, anthracyclines can induce diffuse myocardial fibrosis later, which can be confirmed by elevated MRI T1 times.^33^ However, it has been reported that myocardial MRI T2 times and serum biomarkers were not able to stratify patients and identify those at risk of developing cardiotoxicity.^32^ Therefore, it is obvious that there is an ongoing myocardial remodeling process following treatment with anthracyclines. While there may be T1 reduction shortly post anthracyclines, on the long run myocardial remodeling may lead to elevated T1 times and ECV values because of diffuse interstitial fibrosis in the myocardium.^34^

We conducted a single-center, observational, prospective study to evaluate the value of cardiac MRI parameters for revealing and characterizing cardiac dysfunction associated with anthracyclines in sarcoma and breast cancer patients.

## Patients and methods

All work has been conducted in accordance with the Declaration of Helsinki (1964). The study was approved by our institutional review board (IRB) and informed consent forms were collected from all subjects. Eighteen patients (5 males and 13 females; 8 sarcoma and 10 breast cancer) scheduled for doxorubicin chemotherapy were included in the study. The patients underwent a baseline (pretreatment (18 patients)) and two follow-up (posttreatment completion (14 patients) and 6-months after treatment completion (10 patients)) visits. The treatment duration was 143 ± 65 days. Each visit included an optimized cardiac MRI exam and blood analysis. A questionnaire about risk factors and comorbidities was collected from the patients at the first visit. The MRI exams were conducted on a 3T GE MRI scanner (GE Healthcare, Waukesha, WI, USA)) and included the following sequences: cine, tagging, modified Look-Locker (MOLLI) T1 mapping (both pre and post gadolinium (Gd) injection), T2 mapping, perfusion, and late gadolinium enhancement (LGE).

The acquired cine images included a stack of parallel short-axis slices (SAX) covering the heart from base to apex in addition to 2-chamber, 3-chamber, and 4-chamber long-axis slices. The optimized cine imaging parameters were as follows: fast imaging employing steady-state acquisition (FIESTA) acquisition, repetition time (TR) = 3.6 ms, echo time (TE) = 1.3 ms, flip angle = 55°, views per segment = 14, # averages = 1, matrix = 256 × 256, slice thickness = 8 mm, and readout bandwidth = 488 Hz/pixel. The acquired tagged images included a 3 equidistant short-axis slices (basal, mid-ventricular, and apical) in addition to 2-chamber, 3-chamber, and 4-chamber long-axis slices. Optimized tagging imaging parameters different from cine imaging were as follows: TR = 5.7 ms, TE = 3.1 ms, flip angle = 8°, views per segment = 14, readout bandwidth = 391 Hz/pixel, tag spacing = 7mm, and number of heart phases = 25.

The MOLLI images were acquired at the same 3 SAX tagged slices (basal, mid-ventricular, and apical). Optimized MOLLI imaging parameters different from cine imaging were as follows: 8 images acquired using the 5(3 s)3 acquisition pattern, TR = 2.9 ms, TE = 1.3 ms, flip angle = 35°, and readout bandwidth = 977 Hz/pixel. The T2 mapping images were acquired at the same 3 SAX tagged slices (basal, mid-ventricular, and apical). Optimized T2 mapping imaging parameters different from cine imaging were as follows: multi-echo fast spin echo sequence, TR = 895 ms, TE = 11 − 77 ms (4 echoes with 22 ms increments), echo train length (ETL) = 16, flip angle = 90°, and readout bandwidth = 651 Hz/pixel.

The perfusion images were acquired at the same 3 SAX tagged slices (basal, mid-ventricular, and apical). Optimized perfusion imaging parameters different from cine imaging were as follows: Fast gradient echo (FGRE) acquisition, TR = 2.5 ms, TE = 1.7 ms, flip angle = 20°, inversion time (TI) = 173 ms, ETL = 1, # averages = 0.75, number of multiphase images = 60, and readout bandwidth = 326 Hz/pixel. The LGE images were acquired at the same SAX and LAX cine slices. Optimized LGE imaging parameters different from cine imaging were as follows: TR = 5.1 ms, TE = 2.3 ms, flip angle = 25°, TI = 275 − 325 ms based on Look-Locker images, and readout bandwidth = 139 Hz/pixel.

Image analysis was conducted by a MRI physicist with 18 years of experience in cardiac MRI (E.I.) and independently reviewed by a cardiothoracic radiologist with 13 years of experience (A.S.). The cine images were analyzed using the cvi42 software (Circle, Calgary, Canada) function module to measure EF and myocardial mass. The T1 and T2 images were analyzed using the cvi42 software T1 and T2 mapping modules, respectively, to generate T1, T2, and ECV maps. The SinMod method (InTag, Leon, France) (35) was used to analyze the tagged images to measure myocardial global longitudinal (GLS), circumferential (GCS), and radial (GRS) strains. Finally, the cvi42 software perfusion and tissue characterization modules were used to determine the existence of ischemic perfusion defects and myocardial infarction/scars, respectively. Inter- and intra-observer variabilities of strain analysis using this technique have been previously demonstrated.^35^

The blood samples drawn at each timepoint were analyzed to measure the following biomarkers: N-terminal pro b-type natriuretic peptide (NT-proBNP), Troponin I (TnI), Troponin T (TnT), Interleukin 6 (IL-6), Tumor necrosis factor alpha (TNF*α*), C-reactive protein (CRP), and Galectin 3 (gal-3), as parameters associated with cardiac damage and heart failure.

Statistical analysis was conducted using Excel (Microsoft, Redmond, Washington, USA) and Python (Python Software Foundation, Wilmington, Delaware, USA) to compare measurements pre- and post-treatment or between patient subgroups and to assess correlation between different MRI parameters and dose. When examining serial longitudinal measurements, a one-way analysis of variance (ANOVA) was used. Three groups were formed depending on timepoints: baseline, post-treatment, and 6-months follow-up. The Shapiro-Wilk test and the Levene′s test were performed for normality and homogeneity of variances, respectively. If either of these tests has a p-value less than or equal to 0.05, non-parametric alternatives such as the Kruskal-Wallis H-test was performed. Correlation maps between dose and different post-treatment strain components indicated a need for regional analysis at the LV base, mid-ventricle, and apex levels. Correlation coefficients greater than 0.7 were considered high, indicating high dose-response or segment-response relationships. p values < 0.05 was considered significant.

## Results

The demographic data of all patients as well as of the sarcoma and breast cancer subgroups is shown in Table 1.

**TABLE 1.** Patients’ demographic parameters

Parameter	All	Sarcoma	Breast
Number of patients (m/f)	5/13	4/4	1/9
Number of patients – visit A (baseline)	18	8	10
Number of patients – visit B (post treatment)	14	6	8
Number of patients – visit C (6 months post follow-up)	10	4	6
Age (years)	56 ± 13	56 ± 15	55 ± 12
Body mass index (BMI) (kg/m^2^)	29 ± 6	27 ± 8	31 ± 4
White/Black race (n)	17/1	8/0	9/1
Non-Hispanic / Hispanic (n)	18/0	8/0	10/0
Patients with cardiovascular risk factors (n)	3	1	2
Patients with comorbidities (n)	7	5	2
Patients with cardiovascular disease (n)	1	1	0
Smoker patients (n)	7	3	4
Alcohol consumer patients (n)	8	3	5
Patients receiving cardiac medications (n)	4	3	1
Accumulative Dox dose (mg)	514 ± 190	564 ± 277	469±42

1 Dox = doxorubicin; f = female; m = male

**FIGURE 1.**
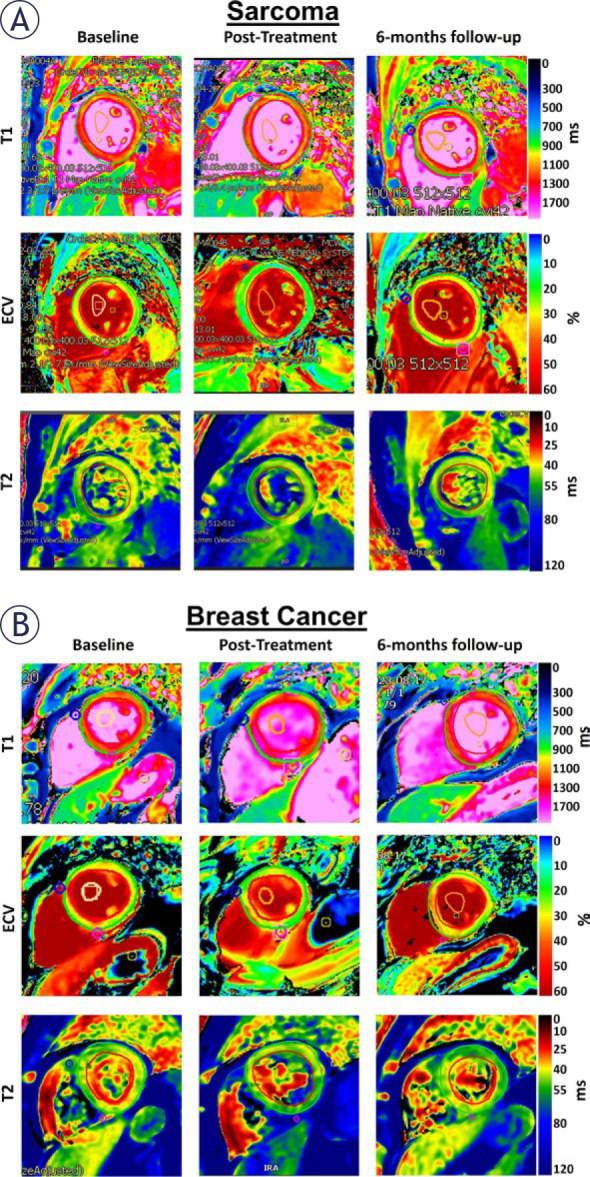
MRI tissue characterization maps in **(A)** sarcoma and **(B)** breast cancer. The figure shows myocardial T1, extracellular volume (ECV), and T2 maps. Note changes in the maps between sarcoma and breast cancer patients as well as between different study timepoints (baseline, posttreatment, 6-months follow-up).

**FIGURE 2.**
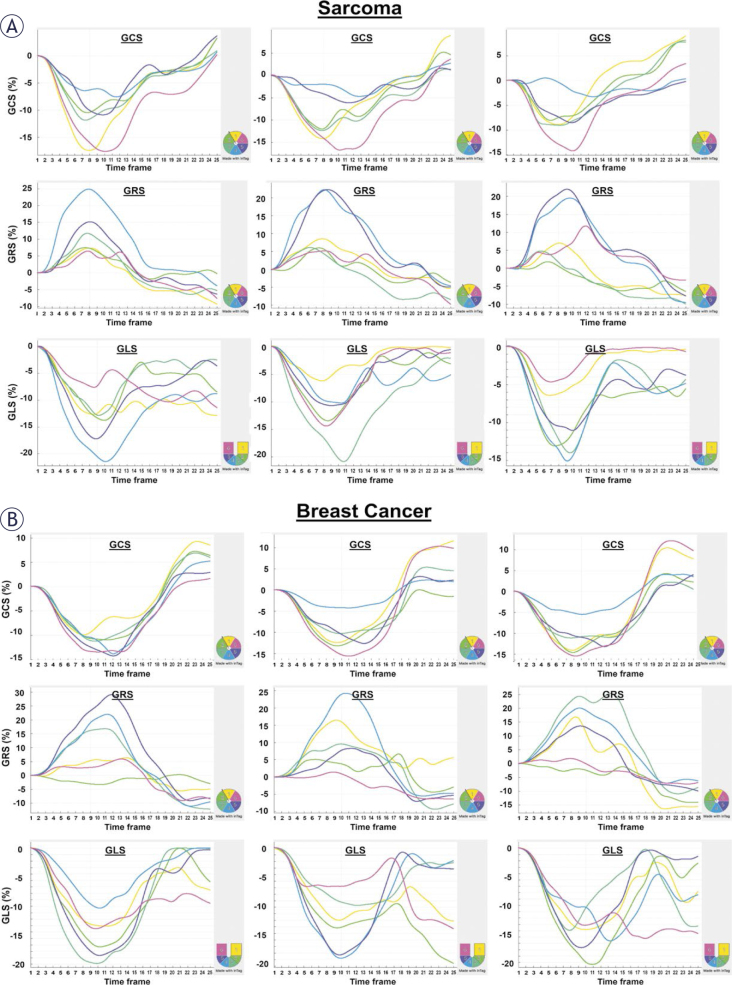
MRI strain curves in **(A)** sarcoma and **(B)** breast cancer patients. The figure shows circumferential (GCS), radial (GRS), and longitudinal (GLS) strain curves in both patient groups at different study timepoints. Myocardial strain for each case is represented by 6 segmental strain curves, color-coded based on the regional location as shown by the lower-right 6-segment illustration based on AHA standard LV model. Note changes in the strain curves between sarcoma and breast cancer patients as well as between different study timepoints (baseline, post-treatment, 6-months follow-up). Note also differences in segmental strain values within the same slice.

Figure 1 and Figure 2 show tissue characteristics and myocardial strains, respectively, in both sarcoma and breast cancer patients at different study timepoints. The MRI measurements for all patients as well as for the sarcoma and breast cancer subgroups at the three timepoints: (A) baseline, (B) post-treatment, and (C) 6-months follow-up are shown in Table 2. One-way ANOVA showed insignificant differences in most MRI parameters between the three timepoints (baseline, post-treatment, and 6-months follow-up). However, it should be noted that all strain values at baseline (pretreatment) were lower than normal strain threshold of 17%, which emphasizes the importance of baseline strain measurements as they reflect underlying risk factors.

**TABLE 2.** Cardiac MRI parameters

Parameter	All	Sarcoma	Breast	p
LVEF (%) - A	59 ± 11	62 ± 10	57 ± 12	0.398
LVEF (%) - B	55 ± 11	57 ± 8	53 ± 14	0.547
LVEF (%) - C	60 ± 5	58 ± 8	61 ± 2	0.489
LV mass (g/m^2^) - A	48 ± 10	48 ± 11	47 ± 9	0.849
LV mass (g/m^2^) - B	46 ± 8	51 ± 9	42 ± 5	0.049*
LV mass (g/m^2^) - C	49 ± 8	53 ± 9	47 ± 7	0.300
RVEF (%) - A	50 ± 10	53 ± 8	48 ± 11	0.256
RVEF (%) - B	45 ± 15	51 ± 11	40 ± 17	0.196
RVEF (%) - C	48 ± 6	50 ± 5	47 ± 7	0.468
GLS (%) - A	-14 ± 2	-14 ± 2	-14 ± 2	0.849
GLS (%) - B	-13 ± 2	-13 ± 2	-14 ± 2	0.272
GLS (%) - C	-14 ± 1	-14 ± 1	-13 ± 1	0.468
GCS (%) - A	-11 ± 2	-11 ± 3	-11 ± 1	0.880
GCS (%) - B	-11 ± 3	-11 ± 3	-11 ± 2	0.733
GCS (%) - C	-12 ± 2	-12 ± 2	-11 ± 2	0.546
GRS (%) - A	11 ± 3	9 ± 3	12 ± 3	0.042*
GRS (%) - B	10 ± 3	9 ± 3	10 ± 2	0.705
GRS (%) - C	10 ± 3	10 ± 3	11 ± 3	0.555
T1 (ms) - A	1264 ± 53	1275 ± 58	1255 ± 50	0.444
T1 (ms) - B	1309 ± 72	1296 ± 48	1319 ± 87	0.543
T1 (ms) - C	1289 ± 97	1213 ± 71	1339 ± 80	0.034*
T2 (ms) - A	49 ± 5	48 ± 5	49 ± 4	0.529
T2 (ms) - B	49 ± 3	48 ± 3	50 ± 3	0.204
T2 (ms) - C	50 ± 3	49 ± 2	52 ± 4	0.136
ECV (%) - A	36 ± 7	37 ± 8	35 ± 6	0.433
ECV (%) - B	35 ± 5	34 ± 6	36 ± 4	0.591
ECV (%) - C	37 ± 4	33 ± 4	39 ± 3	0.031*

1 A, B, and C refer to the three study timepoints (baseline, post-treatment, and 6-months followup), respectively.

1 p values are shown for all measurements. p < 0.05 is considered significant and marked by an asterisk (*).

1 ECV = extracellular volume; EF = ejection fraction; GLS, GCS, GRS = global longitudinal, circumferential, and radial strains, respectively; LV = left ventricle; RV = right ventricle

**FIGURE 3.**
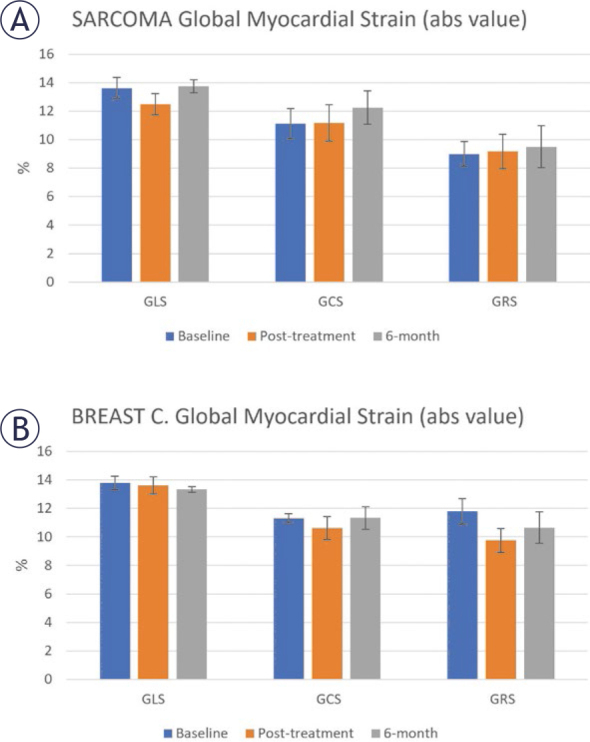
Global longitudinal (GLS), circumferential (GCS), and radial (GRS) strains at baseline, post-treatment, and 6-months follow-up in **(A)** sarcoma and **(B)** breast cancer patients. Note different patterns of change in strain between the two patient groups. In general, GRS is lower than GCS, which is lower than GLS. GCS and GLS are represented by absolute value (original values are negative) for clearer presentation along with positive GRS.

Different strain measurements (GLS, GCS, and GRS) at the three study timepoints in sarcoma and breast cancer patients are shown in Figure 3. GLS and GCS are presented in absolute value (original numbers are negative) for clearer presentation along with the positive GRS. In general, in both patient subgroups, GRS was lower than GCS, which in turn was lower than GLS. In both patient sub-groups, the 6-months follow-up strain were slightly higher than the post-treatment strain, especially for GCS and GRS (p > 0.05 for both groups).

**FIGURE 4.**
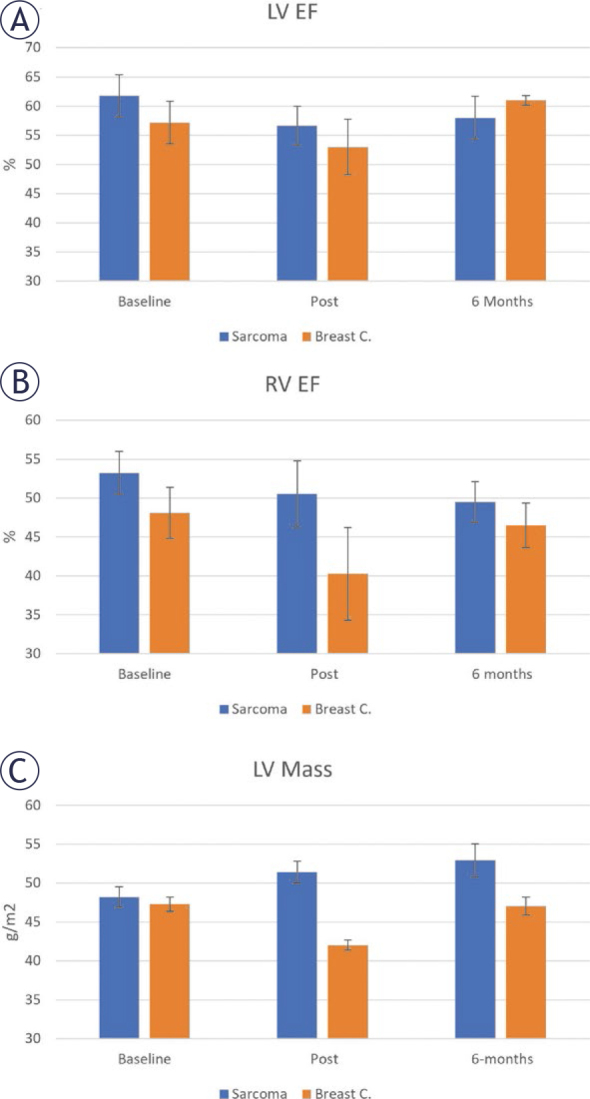
Global cardiac function parameters: **(A)** left ventricular ejection fraction (RVEF), **(B)** right ventricular ejection fraction (RVEF), and (**C**) LV mass at baseline, posttreatment, and 6-months follow-up timepoints in sarcoma and breast cancer patients. LVEF is normal in both groups at all timepoints. RV EF is lower in breast cancer compared to sarcoma. LV mass shows continuous increase with time in sarcoma.

Global measures of cardiac function (LV EF, RV EF, and LV mass) in both sarcoma and breast cancer patients at the three timepoints are shown in Figure 4. LVEF decreased at post-treatment, then increased back at the 6-months follow-up timepoint. LVEF was larger in the sarcoma patients, compared to the breast cancer patients, at both baseline and post-treatment timepoints. However, it was smaller at the 6-months follow-up. RV EF showed slight decrease with time in the sarcoma patients. However, in the breast cancer patients, RV EF showed large decrease at post-treatment before it increased back at the 6-months timepoint. LV mass increased with time in the sarcoma patients, while in the breast cancer patients, it decreased at post-treatment, then increased back at 6-months follow-up. Nevertheless, the changes were not statistically significant (p > 0.05).

**FIGURE 5.**
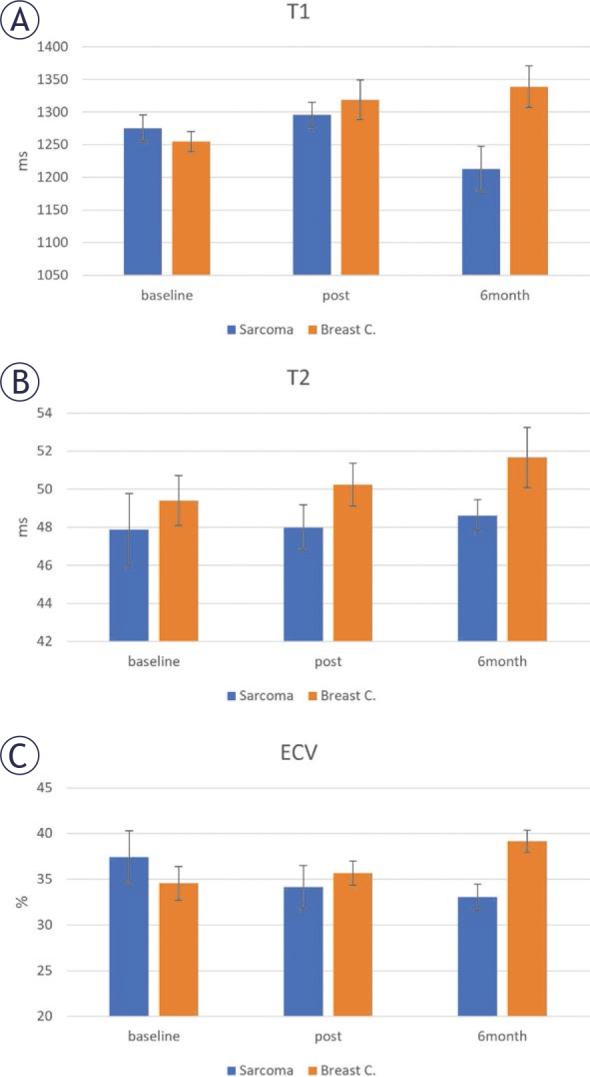
Myocardial **(A)** T1, **(B)** T2, and **(C)** extracellular volume (ECV) measurements in sarcoma and breast cancer groups at different study timepoints. All parameters show continuous increase with time in breast cancer. Sarcoma shows different patterns of change, *e.g*., continuous decrease of ECV with time. Post-treatment and 6-months follow-up T1 values in sarcoma are lower than those in in breast cancer. T2 shows minimal increase with time in sarcoma.

Myocardium tissue characterization (T1, T2, and ECV) measurements in sarcoma and breast cancer patients are shown in Figure 5. The patterns were different between the two patient subgroups. For T1 in sarcoma patients, it slightly increased at posttreatment, then decreased at 6-months follow-up; however, T1 continuously increased with time in the breast cancer patients. For T2 in sarcoma patients, it slightly increased with time; however, it showed larger increase with time in the breast cancer patients. For ECV, it continuously decreased and increased with time in the sarcoma and breast cancer patients, respectively. Perfusion analysis revealed ischemic defects in eleven subjects (5 sarcoma and 6 breast cancer patients), mostly in the basal septal region. LGE revealed scars in five subjects (2 sarcoma and 3 breast cancer patients). There were no significant differences (p > 0.05) in MRI parameters between patients with perfusion defects or scars and those without them.

**FIGURE 6.**
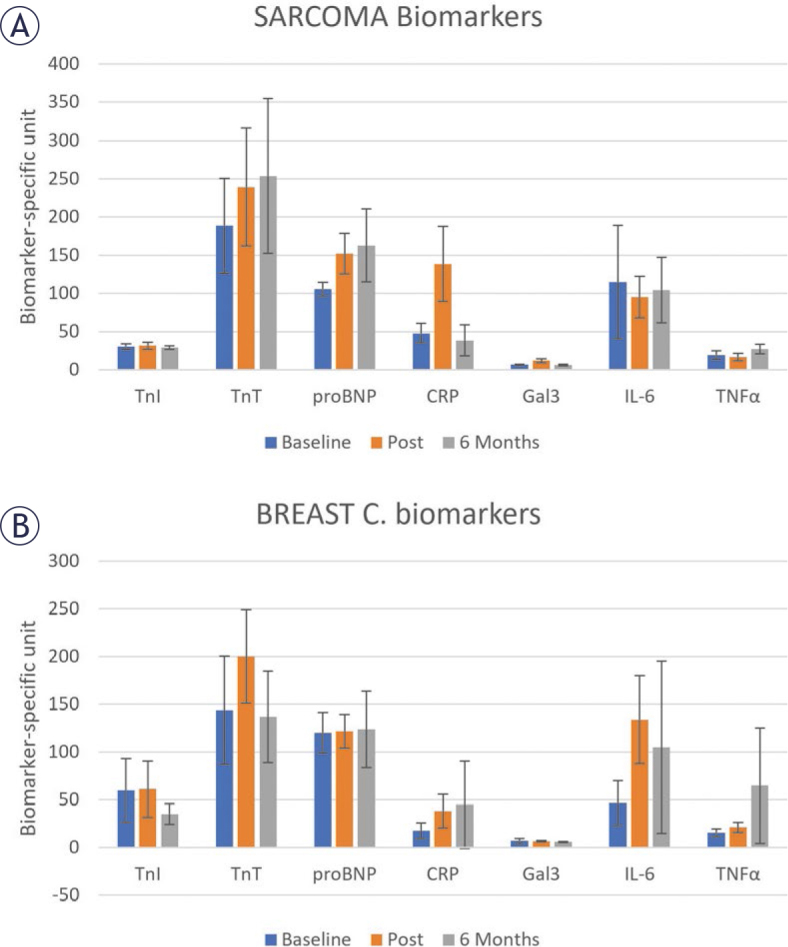
Changes in serum biomarkers at different timepoints in **(A)** sarcoma and **(B)** breast cancer. CRP = C-reactive protein (μg/mL); Gal3 = Galectin 3 (ng/mL); IL-6 = Interleukin 6 (fluorescence intensity units); NT-proBNP = N-terminal pro b-type natriuretic peptide (pg/mL); TNFa = tumor necrosis factor alpha (pg/mL); TnI = cardiac troponin I (florescence intensity units), TnT = cardiac troponin T (pg/mL). The figure shows different patterns of change in the biomarkers between sarcoma and breast cancer. Not all biomarkers increased post-treatment. Different parameters reflect different aspects of cardiac injury.

The patterns of change in the serum biomarkers are different in the two patient groups (Figure 6). In sarcoma patients, both TnT and NT-proBNP showed increase with time, TnI showed similar values at the three timepoints. CRP increased post-treatment then decreased at 6-months follow-up, while IL-6 showed the opposite pattern. Changes in Gal3 and TNF*α* showed small differences between the three timepoints. In breast cancer patients, both CRP and TNF*α* increased with time. NT-proBNP and Gal3 showed similar values at the three timepoints, while TnI, TnT, and IL-6 increased at post-treatment then decreased at 6-months follow-up. TnI and IL-6 are presented in fluorescence intensity (FI) units as the observed values were too small (out-of-range) to report. Differences in serum biomarker measurements were not statistically significant.

**FIGURE 7.**
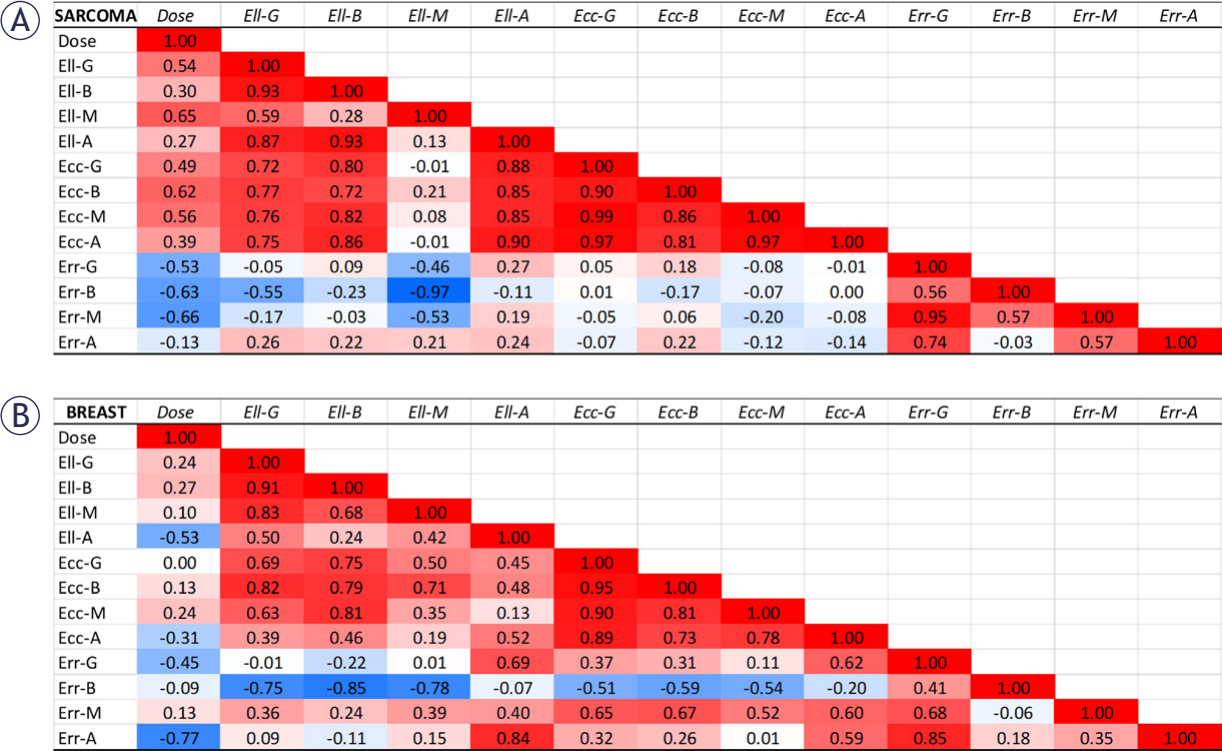
Correlation maps between dose and different post-treatment strain components in the **(A)** sarcoma and **(B)** breast cancer patients at the global level (G) and regional levels (base (B), mid-ventricular (M), and apical (A). Ell, Ecc, and Err represent longitudinal, circumferential, and radial strains, respectively. There is a clear inverse correlation between strain and dose in sarcoma, which is positive for Ell and Ecc and negative for Err, as shown in the leftmost column. Such correlation pattern is not shown in the breast cancer correlation map.

Correlation maps between dose and different strain components at the post-treatment timepoint are shown in Figure 7. The patterns are different for the two patient groups. In sarcoma (Figure 7), as shown in the left-most column, dose is positively correlated with longitudinal (Ell) and circum-ferential (Ecc) strains, while it is negatively correlated with radial (Err) strain. This behavior is consistent for global strain as well as regional values at the base, mid-ventricular, and apical levels. The correlation coefficient values were moderate for all regions except for apical radial strain (last component in the column). These relationships demonstrate that strain gets worse (higher Ell and Ecc and lower Err) with dose, which is expected in cancer patients. However, in the breast cancer patients (Figure 7), the correlation pattern was not consistent and there existed small positive and negative correlations between dose and each of the strain components (Ell, Ecc, Err). The only (negative) high correlation existed between dose and apical radial strain, the opposite case of sarcoma patients. No correlations were statistically significant (p > 0.05).

**FIGURE 8.**
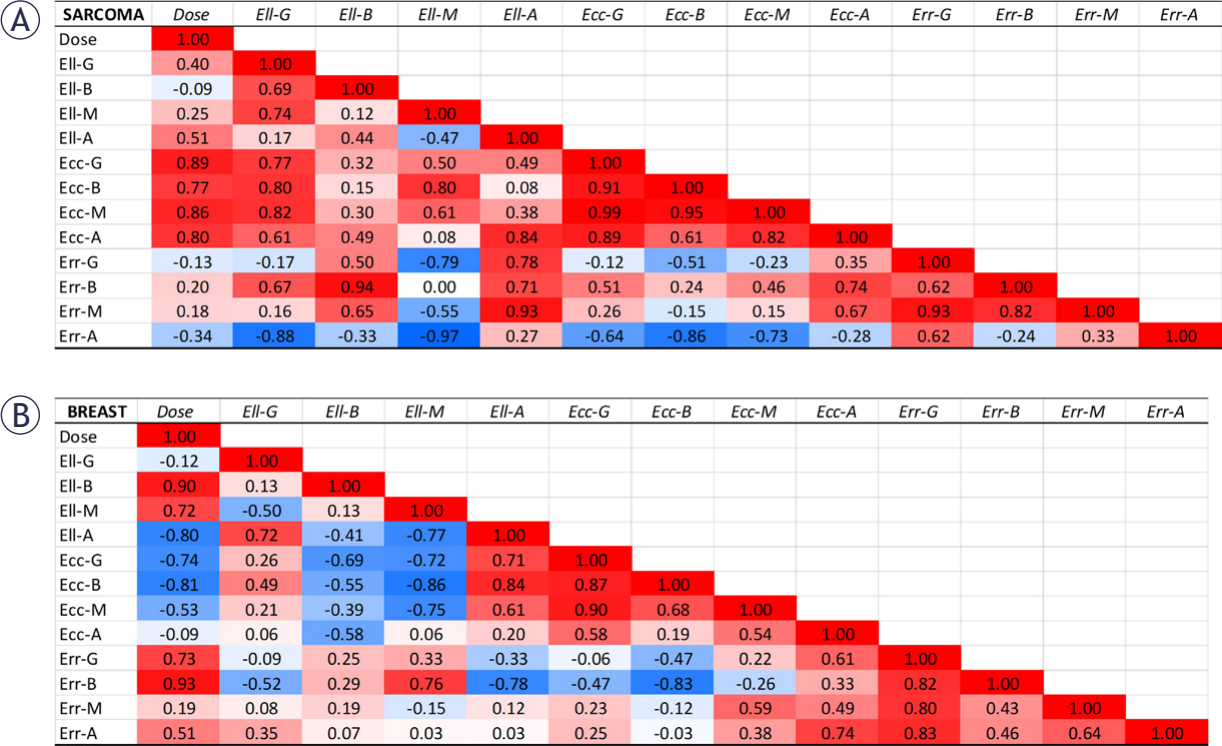
Correlation maps between dose and different 6-months follow-up strain components in the **(A)** sarcoma and **(B)** breast cancer patients at the global level (G) and regional level (base (B), mid-ventricular (M), and apical (A). Ell, Ecc, and Err represent longitudinal, circumferential, and radial strains, respectively. There is inverse correlation only between circumferential strain and dose in sarcoma, which is positive for Ecc. There is inverse correlation only between longitudinal base and mid-ventricular strains and dose in breast cancer, which is positive for Ell-B and Ell-M.

At 6-months follow-up, the correlation patterns changed as shown in Figure 8. In sarcoma patients, the positive correlations between dose and Ecc was maintained, even with higher correlation coefficient compared to the post-treatment timepoint (Figure 7). Significant correlation coefficients existed between dose and both global Ecc (p = 0.017) and mid-ventricle Ecc (0.028), respectively. However, in the breast cancer patients, only basal Ell (p = 0.015) and mid-ventricular Ell (p = 0.107) showed high positive correlations with dose.

In the myocardial segmental level, using AHA 16-segment model (segments 1-6, 7-12, and 13-16 represent basal, mid-ventricular, and apical regions, respectively, in the longitudinal (Ell), circumferential (Ecc), and radial (Err) directions. By taking 0.7 as a threshold for correlation coefficients, the following myocardium segments showed high correlation coefficients (negative for Ell and Ecc and positive for Err) with dose in sarcoma patients at post-treatment: Ell8 (R = 0.71, p = 0.114), Ell9 (R = 0.86, p = 0.028), Ecc1 (R = 0.78, p = 0.067), Ecc7 (R = 0.84, p = 0.036), Ecc9 (R = 0.73, p = 0.099). At 6-months follow-up, the following segments had correlation coefficients above the 0.7 threshold: Ell2 (R = 0.84, p = 0.036), Ell8 (R = 0.82, p = 0.46), Ecc1 (R = 0.74, p = 0.093), Ecc4 (R = 0.79, p = 0.062), Ecc5 (R = 0.75, p = 0.086), Ecc8 (R = 0.9, p = 0.015), Ecc9 (R = 0.75, p = 0.085), Ecc11 (R = 0.70, p = 0.122), Ecc12 (R = 0.85, p = 0.032). In the breast cancer patients, only Err13 (R = -0.89, p = 0.017) and Err14 (R = -0.74, p=0.093) showed high correlation coefficients post treatment. At 6-months, the following segments showed high correlation coefficients: Ell2 (R = 0.95, p = 0.004), Ell5 (R = 0.99, p <0.001), Ell7 (R = 0.98, p <0.001), Ell9 (R = 0.8, p = 0.056), Ell11 (R = 0.75, p = 0.086), Ell14 (R = 0.92, p = 0.009), Ecc8 (R = 0.92, p = 0.009), Err2 (R = -0.92, p = 0.009), Err9 (R = -0.8, p = 0.06), Err14 (R = -0.8, p = 0.06).

## Discussion

The study demonstrates important points about anthracycline-induced cardiotoxicity. The first point is that MRI strain parameters are sensitive biomarkers of cardiac dysfunction (Figure 2). While LVEF was always > 50% in both patient groups and at different study timepoints, strain parameters showed underlying subclinical dysfunction, even at baseline as all strain (absolute) values were less than strain threshold of 17% for normal contractility. Baseline strain parameters inform about underlying risk factors, which should be taken into consideration for patient stratification and prognosis. RVEF (Table 2) in sarcoma was always higher than that in breast cancer, which may be related to the difference in tumor locations and sizes between the two patient groups.

Although previous reports pointed to decreased LV mass post treatment with anthracyclines^32^, this was only demonstrated in the breast cancer patients (Table 2). The opposite pattern occurred in the sarcoma patients where myocardial mass continuously increased post-treatment and at 6-months follow-up. This may represent an undergoing LV remodeling.

The contractility pattern and changes in cardiac MRI parameters were different in sarcoma and breast cancer patients (Figure 3, Table 2). For example, while myocardial strain decreased post-treatment in breast cancer patients, this was not the case in sarcoma. This may imply different mechanisms in response to anthracyclines in breast cancer and sarcoma, especially that sarcomas can have large tumors more often than those in breast cancers, where removing a large tumor would actually result in the body being healthier after treatment.

The changes in myocardial function post-treatment were accompanied by changes in myocardial tissue characterization based on MRI relaxometry maps (Figure 5, Table 2). In breast cancer patients, all T1, T2, and ECV parameters showed continuous increase from baseline to post-treatment to 6-months follow-up, which was not the case in sarcoma. The increases in T1, T2, and ECV reflect increased diffuse fibrosis, edema, and collagen formation, respectively. These results show that breast cancer patients are more affected by changes in tissue composition compared to sarcoma patients.

Changes in serum biomarkers showed inconsistent patterns between different parameters and between the sarcoma and breast cancer patients (Figure 6). For example, only TnT (biomarkers of damage to heart muscle) and NT-proBNP (bio-marker of heart failure) showed continuous increase post-treatment in sarcoma patients versus CRP (biomarker for inflammation) and TNF*α* (biomarker for heart failure) in breast cancer patients. This may reflect different mechanisms in response to anthracyclines in the two patient groups.

Post-treatment myocardial strain (Figure 7) showed good correlation with anthracycline dose in the sarcoma patients (positive correlations with GLS and GCS and negative correlation with GRS), which means deteriorated strain value is associated with higher dose. However, this pattern was not observed in breast cancer patients. At 6-months follow-up (Figure 8), only GCS in sarcoma patients showed an inverse correlation with dose, while basal and mid-ventricular GLS showed inverse correlations in the breast cancer patients. This demonstrates the importance of regional myocardial strain as early marker of cardiac dysfunction that is correlated with dose, especially in sarcoma.

On the segmental level (based on American Heart Association (AHA) 16-segment model of the LV), only few segments showed high correlation coefficients (> 0.7) with dose post-treatment. In sarcoma, these segments are septal mid-ventricular longitudinal strain, anterior circumferential strain, and basal anteroseptal radial strain. In breast cancer patients, only apical anterior and septal segmental strains had correlation coefficient values above the threshold. These results may imply different regional myocardial sensitivity to anthracyclines, a subject that is worth investigation in a separate study by itself.

A few points to be considered about this study. First, one limitation of the study is the small sample size and patients who did not complete the post-treatment or 6-months follow-up timepoints, which did not allow for statistically significant results. The small sample size also reduced the biological variability of our patient population. Although our study was underpowered to be able to detect large deteriorations in cardiac function, the clear trends in the results and differences between the two patient groups warrant a follow-up larger study to confirm these results. Secondly, we used MRI in our study, which is more powerful than CT for examining cardiac function. In our study we used standard 8-mm slice thickness as slice thickness of 8-10 mm is typically used in MRI for functional and tissue characterization analyses of the heart in humans, which does not compromise the in-plane resolution ~ 1-2 mm. It should be noted that reduction in slice thickness is associated with reduced signal-to-noise ratio (SNR), which compromises the results accuracy. However, we use thinner slices (1-mm slice thickness) in our small-animal preclinical work on rats^36^ to avoid partial volume effects in the small heart of the rat and we compensate for small SNR by acquiring multiple averages at the cost of increased scan time. Thirdly, unfortunately, histological analysis was not conducted in the patients in this study as neither biopsy nor surgery was conducted on the patients. However, we have conducted histological analysis on our recent preclinical work^37^, in which we conducted hematoxylin and eosin (H&E), Masson′s trichrome, and toluidine blue staining on a rat model of thoracic cancer. The histopathological results confirmed the findings by the imaging biomarkers, which was demonstrated in the samples by increased fibrosis or collagen (Masson trichrome), hemorrhage, cellular vacuolization, and/or cellular necrosis (H&E), and mast cells (toluidine) in the rat model compared to control. Finally, we used the standard American Heart Association (AHA) 16-segment model on all studied subjects and study timepoints. The model represents segmental distribution of the left ventricle myocardium at three levels (base, mid-ventricle, and apex) and different regions (anterior, inferior, septal, and lateral). This model is extensively used for standard cardiac functional analysis as reported in the literature.^38^ We used the model to represent all strain components (circumferential, longitudinal, and radial) for each segment at baseline, post-treatment, and follow-up timepoints. We used correlation analysis between dose and strain parameters to examine the predictive value of different myocardial segments in response to chemotherapy. The results with high correlation (|R| ≥ 0.7) and especially those with significant measurement (p < 0.05) were considered of more influence and predictivity on the dose-response effect. The results showed different segments for sarcoma vs. breast cancer at both post-treatment and 6-months follow-up timepoints.

In conclusion, cardiac MRI provides valuable information about heart function and changes in tissue composition in sarcoma receiving anthracyclines. Especially, myocardial strain is an early marker of cardiac dysfunction when EF > 50%. The generated MRI parameters may reflect a specific contractility and remodeling pattern in sarcoma that is differentiated from breast cancer.

## References

[1] Quintana RA, Banchs J, Gupta R, Lin HY, Raj SD, Conley A (2017). Early evidence of cardiotoxicity and tumor response in patients with sarcomas after high cumulative dose Doxorubicin given as a continuous infusion. Sarcoma.

[2] Coleman MP, Forman D, Bryant H, Butler J, Rachet B, Maringe C (2011). Cancer survival in Australia, Canada, Denmark, Norway, Sweden, and the UK, 1995-2007 (the International Cancer Benchmarking Partnership): an analysis of population-based cancer registry data. Lancet.

[3] Jones RL, Wagner AJ, Kawai A, Tamura K, Shahir A, Van Tine BA (2021). Prospective evaluation of doxorubicin cardiotoxicity in patients with advanced soft-tissue sarcoma treated in the ANNOUNCE phase III randomized trial. Clin Cancer Res.

[4] Krone RJ, Van Tine BA. (2023). More data to support a cardiac-oncologic partnership. JACC CardioOncol.

[5] Swain SM, Whaley FS, Ewer MS. (2003). Congestive heart failure in patients treated with doxorubicin: a retrospective analysis of three trials. Cancer.

[6] Mo Z, Deng Y, Bao Y, Liu J, Jiang Y. (2023). Evaluation of cardiotoxicity of anthracycline-containing chemotherapy regimens in patients with bone and soft tissue sarcomas: a study of the FDA adverse event reporting system joint single-center real-world experience. Cancer Med.

[7] Paulides M, Kremers A, Stohr W, Bielack S, Jurgens H, Treuner J (2006). Prospective longitudinal evaluation of doxorubicin-induced cardiomyopathy in sarcoma patients: a report of the late effects surveillance system (LESS). Pediatr Blood Cancer.

[8] Shamai S, Rozenbaum Z, Merimsky O, Derakhshesh M, Moshkovits Y, Arnold J (2020). Cardio-toxicity among patients with sarcoma: a cardio-oncology registry. BMC Cancer.

[9] Mitani I, Jain D, Joska TM, Burtness B, Zaret BL. (2003). Doxorubicin cardiotoxicity: prevention of congestive heart failure with serial cardiac function monitoring with equilibrium radionuclide angiocardiography in the current era. J Nucl Cardiol.

[10] Altena R, Perik PJ, van Veldhuisen DJ, de Vries EG, Gietema JA. (2009). Cardiovascular toxicity caused by cancer treatment: strategies for early detection. Lancet Oncol.

[11] Bonita R, Pradhan R. (2013). Cardiovascular toxicities of cancer chemotherapy. Semin Oncol.

[12] Khawaja MZ, Cafferkey C, Rajani R, Redwood S, Cunningham D. (2014). Cardiac complications and manifestations of chemotherapy for cancer. Heart.

[13] Yeh ET, Tong AT, Lenihan DJ, Yusuf SW, Swafford J, Champion C (2004). Cardiovascular complications of cancer therapy: diagnosis, pathogenesis, and management. Circulation.

[14] Alvarez JA, Russell RR. (2017). Cardio-oncology: the nuclear option. Curr Cardiol Rep.

[15] Al-Batran SE, Meerpohl HG, von Minckwitz G, Atmaca A, Kleeberg U, Harbeck N (2006). Reduced incidence of severe palmar-plantar erythrodysesthesia and mucositis in a prospective multicenter phase II trial with pegylated liposomal doxorubicin at 40 mg/m2 every 4 weeks in previously treated patients with metastatic breast cancer. Oncology.

[16] Alhaja M, Chen S, Chin AC, Schulte B, Legasto CS. (2023). Cardiac safety of pegylated liposomal doxorubicin after conventional doxorubicin exposure in patients with sarcoma and breast cancer. Cureus.

[17] Mehta LS, Watson KE, Barac A, Beckie TM, Bittner V, Cruz-Flores S (2018). Cardiovascular disease and breast cancer: where these entities intersect: a scientific statement from the american heart association. Circulation.

[18] Shantakumar S, Olsen M, Vo TT, Norgaard M, Pedersen L. (2016). Cardiac dysfunction among soft tissue sarcoma patients in Denmark. Clin Epidemiol.

[19] Ettinghausen SE, Bonow RO, Palmeri ST, Seipp CA, Steinberg SM, White DE (1986). Prospective study of cardiomyopathy induced by adjuvant doxorubicin therapy in patients with soft-tissue sarcomas. Arch Surg.

[20] Cardinale D, Iacopo F, Cipolla CM. (2020). Cardiotoxicity of anthracyclines. Front Cardiovasc Med.

[21] Leger K, Slone T, Lemler M, Leonard D, Cochran C, Bowman WP (2015). Subclinical cardiotoxicity in childhood cancer survivors exposed to very low dose anthracycline therapy. Pediatr Blood Cancer.

[22] Alpman MS, Jarting A, Magnusson K, Manouras A, Henter JI, Broberg AM (2023). Longitudinal strain analysis for assessment of early cardiotoxicity during anthracycline treatment in childhood sarcoma: a single center experience. Cancer Rep (Hoboken).

[23] Heemelaar JC, Speetjens FM, Al Jaff AAM, Evenhuis RE, Polomski EAS, Mertens BJA (2023). Impact of age at diagnosis on cardiotoxicity in high-grade osteosarcoma and Ewing sarcoma patients. JACC CardioOncol.

[24] Tian Z, Yang Y, Yang Y, Zhang F, Li P, Wang J (2020). High cumulative doxorubicin dose for advanced soft tissue sarcoma. BMC Cancer.

[25] Ehrhardt MJ, Leerink JM, Mulder RL, Mavinkurve-Groothuis A, Kok W, Nohria A (2023). Systematic review and updated recommendations for cardiomyopathy surveillance for survivors of childhood, adolescent, and young adult cancer from the International Late Effects of Childhood Cancer Guideline Harmonization Group. Lancet Oncol.

[26] Yoon GJ, Telli ML, Kao DP, Matsuda KY, Carlson RW, Witteles RM. (2010). Left ventricular dysfunction in patients receiving cardiotoxic cancer therapies are clinicians responding optimally?. J Am Coll Cardiol.

[27] Plana JC, Galderisi M, Barac A, Ewer MS, Ky B, Scherrer-Crosbie M (2014). Expert consensus for multimodality imaging evaluation of adult patients during and after cancer therapy: a report from the American Society of Echocardiography and the European Association of Cardiovascular Imaging. J Am Soc Echocardiogr.

[28] Celutkiene J, Pudil R, Lopez-Fernandez T, Grapsa J, Nihoyannopoulos P, Bergler-Klein J (2020). Role of cardiovascular imaging in cancer patients receiving cardiotoxic therapies: a position statement on behalf of the Heart Failure Association (HFA), the European Association of Cardiovascular Imaging (EACVI) and the Cardio-Oncology Council of the European Society of Cardiology (ESC). Eur J Heart Fail.

[29] Oikonomou EK, Kokkinidis DG, Kampaktsis PN, Amir EA, Marwick TH, Gupta D (2019). Assessment of prognostic value of left ventricular global longitudinal strain for early prediction of chemotherapy-induced cardiotoxicity: a systematic review and meta-analysis. JAMA Cardiol.

[30] Lyon AR, Lopez-Fernandez T, Couch LS, Asteggiano R, Aznar MC, Bergler-Klein J (2022). 2022 ESC Guidelines on cardio-oncology developed in collaboration with the European Hematology Association (EHA), the European Society for Therapeutic Radiology and Oncology (ESTRO) and the International Cardio-Oncology Society (IC-OS). Eur Heart J.

[31] Suerken CK, D’Agostino RB, Jordan JH, Melendez GC, Vasu S, Lamar ZS (2020). Simultaneous left ventricular volume and strain changes during chemotherapy associate with 2-year postchemotherapy measures of left ventricular ejection fraction. J Am Heart Assoc.

[32] Muehlberg F, Funk S, Zange L, von Knobelsdorff-Brenkenhoff F, Blaszczyk E, Schulz A (2018). Native myocardial T1 time can predict development of subsequent anthracycline-induced cardiomyopathy. ESC Heart Fail.

[33] Jordan JH, Vasu S, Morgan TM, D’Agostino RB, Melendez GC, Hamilton CA (2016). Anthracycline-associated T1 mapping characteristics are elevated independent of the presence of cardiovascular comorbidities in cancer survivors. Circ Cardiovasc Imaging.

[34] Neilan TG, Coelho-Filho OR, Shah RV, Feng JH, Pena-Herrera D, Mandry D (2013). Myocardial extracellular volume by cardiac magnetic resonance imaging in patients treated with anthracycline-based chemotherapy. Am J Cardiol.

[35] Ibrahim EH, Stojanovska J, Hassanein A, Duvernoy C, Croisille P, Pop-Busui R (2018). Regional cardiac function analysis from tagged MRI images. Comparison of techniques: Harmonic-Phase (HARP) versus Sinusoidal-Modeling (SinMod) analysis. Magn Reson Imaging.

[36] Ibrahim EH, Baruah D, Budde M, Rubenstein J, Frei A, Schlaak R (2020). Optimized cardiac functional MRI of small-animal models of cancer radiation therapy. Magn Reson Imaging.

[37] Ibrahim EH, Baruah D, Croisille P, Stojanovska J, Rubenstein JC, Frei A (2021). Cardiac magnetic resonance for early detection of radiation therapyinduced cardiotoxicity in a small animal model. JACC CardioOncol.

[38] Cerqueira MD, Weissman NJ, Dilsizian V, Jacobs AK, Kaul S, Laskey WK (2002). Standardized myocardial segmentation and nomenclature for tomographic imaging of the heart. A statement for healthcare professionals from the Cardiac Imaging Committee of the Council on Clinical Cardiology of the American Heart Association. Circulation.

